# Delayed hyperbaric oxygen therapy for air emboli after open heart surgery: case report and review of a success story

**DOI:** 10.1186/s13019-016-0553-5

**Published:** 2016-12-05

**Authors:** Eva Niyibizi, Guillaume Elyes Kembi, Claude Lae, Rodrigue Pignel, Tornike Sologashvili

**Affiliations:** 1 Division of Emergency Medecine, County Hospital, University of Geneva, Geneva, Switzerland; 2 Department of Anesthesiology Pharmacology and Intensive Care, County Hospital, University of Geneva, Geneva, Switzerland; 3 Department of Emergency and Primary Care Medecine, County Hospital, Hyperbaric Center, University of Geneva, Geneva, Switzerland; 4 Department of Emergency and Primary Care Medicine, County Hospital, Hyperbaric Center, University of Geneva, Geneva, Switzerland; 5 Division of Cardiac Surgery, County Hospital, University of Geneva, Geneva, Switzerland; 6 Emergency Medicine Division, Hopitaux Universitaires de Genève, Rue Gabrielle-Perret.Gentil 4, 1205 Geneva, Switzerland

**Keywords:** Iatrogenic cerebral air emboli, Neurologic deficit, Cardiopulmonary bypass (CPB), Cardiac surgery, Hyperbaric oxygen therapy (HOT)

## Abstract

**Background:**

The current case describes a rare diagnosis of iatrogenic air emboli after elective cardiopulmonary bypass that was successfully treated with delayed hyperbaric oxygen therapy, with good clinical evolution in spite of rare complications.

**Case presentation:**

A 35 years old male was admitted to the intensive care unit (ICU) for post-operative management after being placed on cardiopulmonary bypass (CPB) for an elective ventricular septal defect closure and aortic valvuloplasty. The patient initially presented with pathologically late awakening and was extubated 17 h after admission. Neurologic clinical status after extubation showed global aphasia, mental slowness and spatio-temporal disorientation. The injected cerebral CT scan was normal; the EEG was inconclusive (it showed metabolic encephalopathy without epileptic activity); and the cerebral MRI done 48 h after surgery showed multiple small subcortical acute ischemic lesions, mainly on the left fronto- parieto- temporo-occipital lobes. He was taken for hyperbaric oxygen therapy (HOT) over 54 h after cardiac surgery. The first session ended abruptly after 20 min when the patient suffered a generalised tonico-clonic seizure, necessitating a moderately rapid decompression, airway management, and antiepileptic treatment. In total, the patient received 7 HOT sessions over 6 days. He demonstrated full neurological recovery at 4 weeks and GOS (Glasgow Outcome Scale) of 5 out of 5 even after a long delay in initial management. Convulsions are a rare complication of HOT either due to reperfusion syndrome or hyperoxic toxicity and can be managed. Prior imaging by MRI or tympanic paracentesis (myringotomy) should not add further delay of treatment.

**Conclusion:**

HOT should be initiated upon late awakening and/or neurologic symptoms after CPB heart surgery, after exclusion of formal counter-indications, even if the delay exceeds 48 h.

## Background

Iatrogenic air emboli is a 0.1% complication of CPB, largely under-diagnosed and under-reported with high morbidity (13–71%) and mortality (5–23%). Cerebral Arterial Air Embolism is a clinical diagnosis; its risk factors should be known and a high index of suspicion should be maintained. HOT should be considered and initiated in the absence of formal counter-indications, even if the delay is over 48 h.

## Case presentation

A 35 year old male was admitted to the hospital for elective cardiovascular surgery for a congenital Ventricular Septal Defect with a moderate left-right shunt and aortic insufficiency, also known as the Laubry-Pezzi Syndrome. He was diagnosed at age 25. Relevant medical history included severe penicillin allergy, type 2 diabetes, hypertension, dyslipidemia, and history of endocarditis of the pulmonary valve with resultant moderate pulmonary valve insufficiency.

The elective surgery consisted of a closure of the ventriculo-septal defect (VSD) and aortic valvuloplasty, under cardiopulmonary bypass (CPB). The whole operative procedure lasted five hours and thirty-five minutes, with aortic clamping duration of one hour and nine minutes, and extracorporeal circulation time of one hour and twenty-nine minutes. The patient underwent combined general and epidural anesthesia. Perioperatively he was hemodynamically stable, and weaning from CPB was uneventful. The transoesophageal echocardiography (TOE) performed by the anesthesiologist after the de-clamping of the aorta, however, showed a significant amount of residual air bubbles, which were then immediately extracted through the aortic canula until complete disappearance.

The patient was transferred to our multidisciplinary intensive care unit around 5 pm right after the surgery. Upon his arrival, he was still intubated but not sedated. The primary clinical examination showed GCS of 11, bradypnea, poor vigilant state, both pupils constricted in myosis but no neurologic focal deficit. His first arterial blood gas showed a respiratory acidosis. All of these findings were consistent with opioid impregnation or a slow metabolic clearance of anesthetic agents. His conscious state and frequents apneas required him to stay on mechanical ventilation with assisted and controlled mode until the end of the night.

On day 1 he was extubated and his neurological examination showed slowness, spatio-temporal disorientation, severe motor and sensory aphasia, no response to simple orders, and no limb deficit. The brief neuropsychological examination in his native tongue at his bedside with a certified interpreter confirmed similar findings. At that stage, the differential diagnosis was a cerebral vascular event - ischemic or haemorrhagic, epilepsy, or an air emboli. The hypothesis of an air emboli was discussed with the operating surgeon and the anesthesiologist, who both confirmed that during the procedure there had been a considerable amount of air bubbles after decanulation of the aorta, an occasional event occurring with CPB; however, no additional transoesophageal echocardiograpy was performed in the ICU at that time.

An injected cerebral CT scan was performed and excluded a recent ischemic attack or secondary bleeding; all arteries were patent. After neurologist consultation, the patient was treated for a small vascular ischemic attack, based on the sole clinical diagnosis, and without specific radiographic findings. He received intravenous aspirin (250 mg) and appropriate hemodynamic monitoring.

**Fig. 1:**
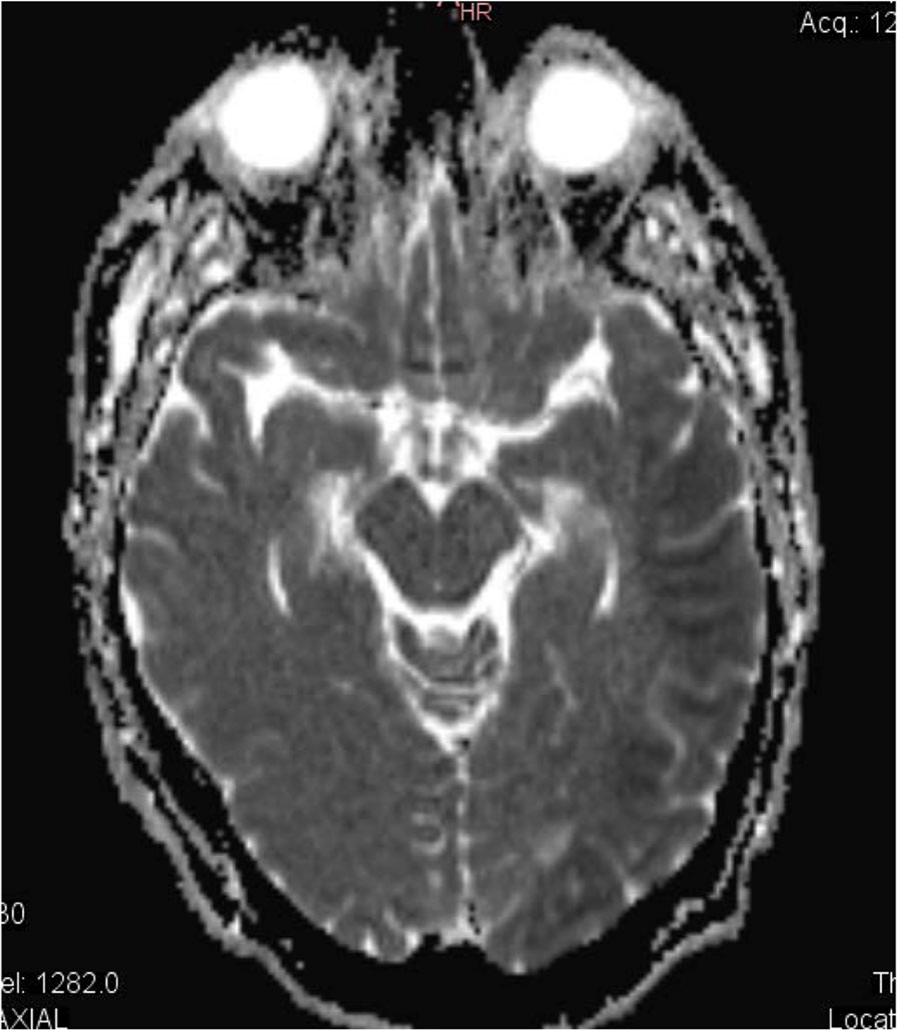
MRI diffusion sequences showing restricted diffusion in the left temporal parietal and occipital areas

**Fig. 2:**
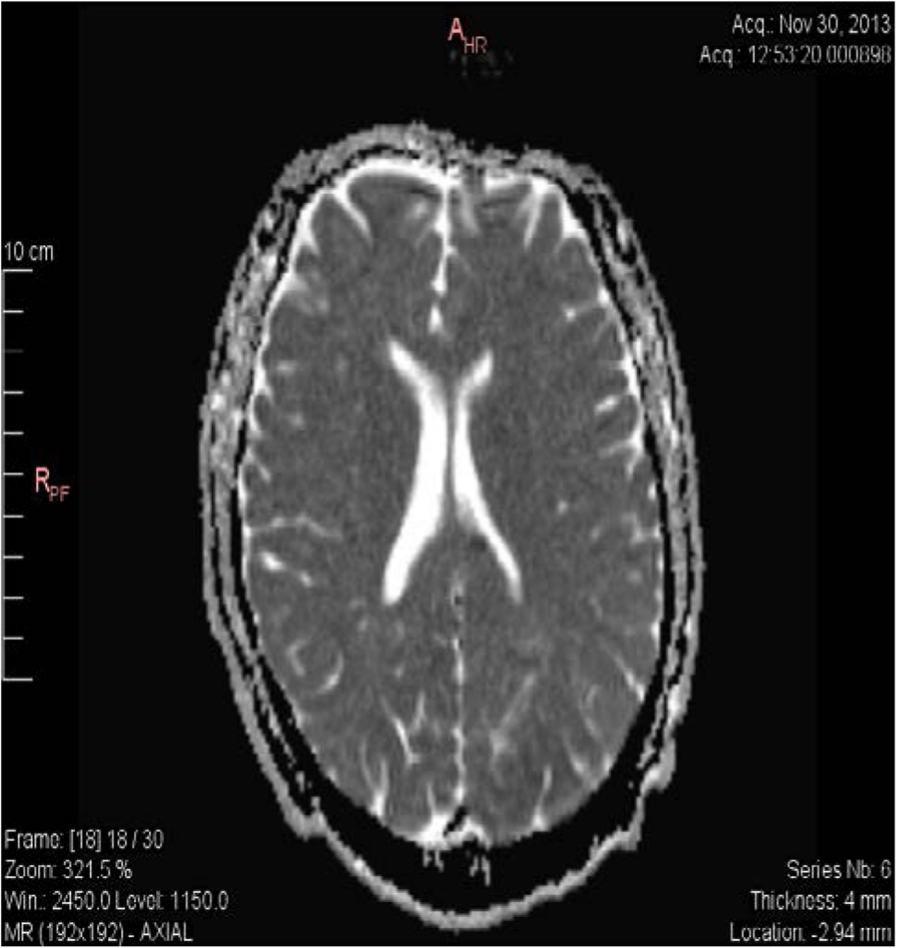
MRI showing small punctiform lesions in subcortical areas of ischemic origin

On day 2, an MRI showed restricted diffusion and cortical lesions (Fig. 1) in the left temporal-parietal and occipital areas, and additional multiple small punctiform lesions in the subcortical areas (in frontiers territories) (Fig. 2) left frontal superior, left precentral, and two other bilateral milimetric lesions on the posterior part of the body of the corpus callosum. All of these small lesions were compatible with acute ischemic lesions. However the small size of the lesions was not consistent with the loud clinical status and a potential subclinical status epilepticus was evoked. The EEG done on day 2 excluded the presence of epileptiform activity and showed a slow tracing compatible with metabolic versus toxic encephalopathy, a nonspecific finding in ICU patients; but a definitive diagnosis could not be confirmed.

**Fig. 3:**
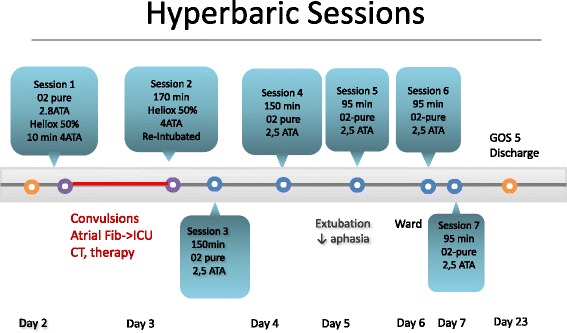
Timeline of Hyperbaric Sessions

**Fig. 4:**
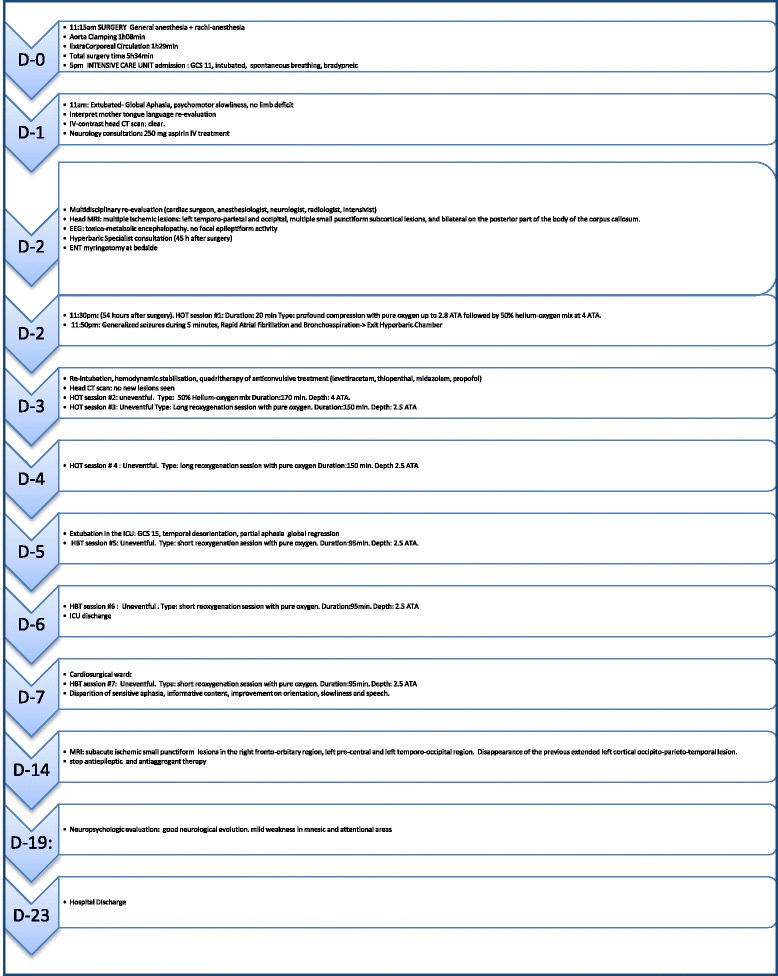
Timeline of Events

Because of the pathologic awakening, persistent neurologic deficit of global aphasia, recent open heart surgery, and the results of outside clinical investigations, we decided collegially to reconsider the possible diagnosis of an air emboli, and assess for hyperbaric oxygen therapy. Luckily, our university medical center in Geneva possesses a renovated hyperbaric chamber with a medical team available 24/7. We contacted them to get their expert opinion, and considering the delay of over 45 h but of less than 72 h, they agreed to take the patient for a few sessions over 3–4 days in the hyperbaric chamber since there was no known contraindications (i.e: undrained pneumothorax, cardiac failure, uncontrolled hypertension, untreated epilepsy and acute severe asthma). The patient entered the hyperbaric chamber accompanied by an intensive care resident doctor, an anesthesiologist and a nurse at 11:30 pm (day2); i.e. 54 h after surgery.

The initial plan (Fig. 3) was to do a first profound compression session CX30 of 7 h and 30 min, starting the first 10 min with pure oxygen up to 2.8 ATA in order to crush the gaz bubble; followed by with a 50% helium-oxygen mix at 4 ATA for the rest of the session. The compression went smoothly. However 20 min after the beginning of the session, at a level of 4 ATA the patient suffered a generalized tonico-clonic seizure, followed by new onset rapid atrial fibrillation and massive bronchoaspiration requiring a moderately rapid decompression, interruption of the session and evacuation of the patient from the chamber. The patient was transferred back to the ICU for stabilisation, airway management with re-intubation and sedation, hemodynamic stabilisation and antiepileptic treatment with adequate therapy (propofol, midazolam, clonazepam, levetiracetam). Another cerebral CT scan did not show any new lesion. After stabilisation, he was taken back to the hyperbaric chamber and the rest of the session (170 min with 50% helium-oxygen mix at 4 ATA) went without any new complications. He suffered a new episode of focal convulsion of the left superior arm one hour after his return from the session that necessitated the adjunction of thiopenthal before resolution.

He received a third session of HOT later (day 3) under mechanical ventilation during 150 min at 2.5 ATA with pure oxygen. On day 4, he also received a 150 min session of HOT while still intubated. He was extubated on day 5, and for the next 2 days the patient underwent each day (day 5-6-7) 1 short session of 95 min under simple mask at 2.5 ATA. In total, the full treatment of HOT consisted of 7 sessions over 5 days.

Except for the adverse events of the first session, all of the remaining sessions went smoothly without complication. The patient recovered rapidly, and was transferred out of the intensive care unit into the cardiovascular surgery ward 6 days after admission. The EEG done at day 5 showed no epileptic activity and the antiepileptic agents were stopped after the HOT sessions were resumed. After the 7th session of HOT at day 7, the patient’s neurological status showed a slight residual spatio-temporal orientation, slowness of speech and a moderate loss of words but his language was informative and he showed no more comprehensive deficit. Considering the good neurological response and improvement on orientation, speech, and psychomotor slowness, HOT sessions were resumed.

On day 14, a control cerebral MRI was done and showed subacute ischemic small punctiform lesions in the right fronto-orbitary region, left pre-central and left temporo-occipital region. The previous extended left cortical occipito-parieto-temporal lesion had disappeared, and was probably due to vasogenic edema. The neurovascular specialists and neuroradiologists concluded that the subcortical and bilateral corpus callosum punctiform lesions were highly compatible with air emboli lesions and therefore there was no indication to pursue aspirin medication upon discharge.

On day 19, a neuropsychological evaluation in English was done, although not in his native tongue, and showed good neurological recovery without pathological sequellae compared to the adult reference population. Detailed examination revealed no orientation disorders. The behavioural analysis showed a slight psychomotor slowness with a lack of task initiation, a discrete lack of verbal spontaneity in the executive area, and borderline memory tests results showing a slight weakness in the episodic, verbal and visuo-spatial memory. There were no language, calcul, or graphic deficits, no speech impairment, and no sign of corpus callousum defect. All of these findings showed mild weaknesses in the mnesic, executives and attentional areas which were compatible with the MRI lesions.

Figure (Fig. 4) summarizes the Timeline of events as they occured sequentially. The patient was discharged from the hospital on post-operative day 23 in good clinical condition, with a neurologic follow up scheduled 8 weeks later. Upon discharge, the Glasgow Outcome Score was of 5 out of 5. (*GOS 1*: death. *GOS 2*: vegetative state with no obvious cortical function. *GOS 3*: severe disability –conscious but disabled with dependency. *GOS 4:* moderate disability but independent. *GOS 5*: complete recovery with resumption of activity, although the patient might show some minor neurological psychological deficit).

Over 5 months after his surgery, a follow-up phone call was made. The patient reported a good recovery. He had mild overall fatigue and had not yet gone back to work, but was without neurological deficit.

## Discussion and literature review

Open heart cardiac surgery with extracorporeal circulation is one of the known causes of air embolism [1] most of the time by entry of air into the extracorporeal bypass pump circuit directly into the systemic circulation, or into the pulmonary veins in case of paradoxical embolism, or in case of incomplete removal of the air from the heart after cardiac arrest [2, 3].

The precise incidence of iatrogenic cerebral arterial air embolism is unknown mostly due to underdiagnosis, underestimation, underreported cases, and seldom non-specific or transient symptomatology [4, 5]. Some previous authors estimated the incidence of 0.1% of all extracorporeal circulation [6]. In 2010, the risk was estimated at 2.6/100 000 procedures. However, although rare, it compasses a prolonged hospital stay with a high morbidity (13–71%), and a high mortality (5–23%) [7].

There are a few common risk factors for post cardiac surgical stroke, such as age, hypertension, diabetes, and previous history of vascular ischemic attack [8–13]. However, there is some evidence that procedures with cardio-pulmonary bypass are more at risk of stroke as opposed to “beating heart” cardiac surgery [14–17]. Valvular replacement surgery is also a greater risk since the ventricle is opened as opposed to coronary artery bypass grafting [8, 11, 12, 18]. Along that line, another risk factor described is any surgery in which the left ventricle and/or the aorta is opened [5].

In a brief reminder of the pathophysiology of cerebral arterial air embolism, we learn that any arterial gas bubble with a size over 30–60um can obstruct the circulation and cause damage through various mechanisms: irritation of the endothelium, cell injury and intra neuronal oedema, inflammation causing vasogenic oedema, and tissue hypoxia. All of these processes will lead to a deficit of perfusion and distal ischemia [2].

Over the last 30 years, many efforts have been made to increase the number of prospective and clinical studies. Unfortunately, due to technical and ethical restrictions and the hyperbaric centers accessibility there isn’t a clear level of scientific evidence [7].

In 2012, Expert Consensus Societies in Europe (ECHM: European Committee for Hyperbaric Medicine) and in America (UHMS: Undersea and Hyperbaric Medical Society) published recommendations recognizing Iatrogenic Symptomatic Air Emboli as one of the four indications for urgent treatment by HOT [7].

In case of iatrogenic symptomatic air emboli, HOT is an adjunct treatment, recommended after conventional treatment of patient positioning, evacuation of air, 100% oxygenation, retrograde perfusion, optimisation of cerebral blood flow, and supportive ventilation measures and/or vasopressors if needed.

Experimental studies showed that delay in initiating HOT was inversely related to its efficacy [19]. Benefits were shown, however, even if the treatment started 12 h after the ischemia when it was a single session treatment [20], and 24 h for a daily session during a week of treatment [21]. One can then think that the delay in initiating the HOT could be partially reversed by increasing the number of sessions administered.

Although there is no level of evidence 1A for HOT use in iatrogenic symptomatic arterial air embolism due to ethical reasons [22], there are some indirect proofs of efficacy since two studies of respectively 86 and 119 patients showed a better recuperation prognosis when HOT is done within the first 6–7 h after the incident [7, 23].

In a 22-year retrospective review of 36 patients who sustained cerebral arterial gas embolism with acute neurologic deficit and were treated with HOT, over 72% patients improved after 24 h. A short (less than 6 h) time to treatment and a young age (less than 45 years) were the two most favourable prognostic factors. The two least favourable prognostic factors were oedema or infarct on the cerebral CT at 24 h after HOT, and the association of cardiopulmonary symptoms [24].

The diagnosis of postoperative iatrogenic cerebral arterial gas emboli is difficult, mostly due to several confounding factors: general anesthesia limiting or delaying a precise neurological examination, cerebral imaging showing different or little results, and low index of suspicion from the medical team.

Based on the literature review, we can conclude that the patient discussed here, although younger than 45 years old, had many risk factors for post-operative stroke (and iatrogenic cerebral arterial embolism): hypertension, diabetes, cardiopulmonary bypass and valvuloplasty.

Research shows that the results of cerebral imaging by CT or MRI in a patient presenting with a neurological deficit after cardiac surgery are often nonspecific and do not allow the definitive diagnosis or exclusion of cerebral arterial gas emboli, and thus should not extend the delay for initiating HOT [2, 4, 25, 26].

In the initial management of this patient, some of the type A medical thinking errors were made on clinical assumptions or tunnel vision, provoking a significant delay in the management of air emboli. The two main reasons we self-critically observed are: first, we had a low index of suspicion, probably due to its very low prevalence; therefore, we wasted time excluding and confirming other diagnoses. Secondly, we thought that even if it was indeed an air emboli, the delay was already too long and therefore no treatment such as HOT could be effective.

In the risk/benefit analysis of HOT, a few practicalities must be considered: the patient’s stability, the availability and accessibility of the nearest HOT center, and the absence of contraindications. However, many hyperbaric medicine centers such as ours can now manage intubated and sedated patient equipped with up to two continuous IV pumps, along with a one-on-one intensive and/or anesthesiology specialised caregiver.

The main risks of the hyperbaric oxygen therapy are rare, and are due to oxygen toxicity on the cardiovascular and neurologic systems. Hyperoxic convulsions are exceptionally rare and due to the reperfusion phenomenon. They usually resolve after stopping the oxygen administration.

In order to avoid unexpected tympanic membrane perforation, our patient had a preventive myringotomy due to his inability to follow simple commands; however this procedure is not mandatory and should not extend the delay to start HOT sessions.

Based on our multidisciplinary experience as intensivist, anesthesiologist, cardiac surgeon, hyperbaric specialist, and acute care, these are the main criteria that we recommend for advising hyperbaric oxygen therapy:

1. Any medical or surgical procedure considered at risk (extracorporeal circulation, central lines manipulation, coelioscopy with high abdominal pressure, open heart-surgery, seated position neurosurgery.)
2. Perioperative or postoperative neurologic symptom or deficit of any nature, including a late awakening after general anesthesia
3. Accessibility to the hyperbaric chamber
4. Patient stability
5. A well trained team of intensivists or anesthesiologists, provided with the right equipment to care for the intubated and sedated patient while in the hyperbaric chamber
6. Absence of contraindications (i.e: undrained pneumothorax, cardiac failure, uncontrolled hypertension, untreated epilepsy and acute severe asthma)

As stated above, radiographic proof or documentation is not mandatory before initiating HOT, since air emboli is first and foremost a clinical diagnosis, and the neuro-radiologic findings are nonspecific in the early hours after the event.

Finally, little is described in the literature regarding the natural history of iatrogenic cerebral arterial air embolism. One may argue that with such a delay of over 54 h prior to HOT, the successful evolution of the patient with complete recovery at 30 days (Glasgow Outcome Scale of 5 out of 5) could be attributed to the natural evolution of the disease, and not so much to the HOT. However, the frequent natural outcome of iatrogenic gas bubble is to be rapidly removed by the pulmonary filters. In our experience, when that is the case, the arterial emboli is eliminated within a few hours with a spontaneous favourable outcome. Unfortunately, if the bubble stays trapped for a period longer than 24 or 36 h, the apoptosis mechanisms are already taking place and necrosis of the cerebral tissue occurs, with risks of sequelae comparable to any other blood clot causing an ischemic cerebro-vascular event.

The fact that our patient’s neurologic deficit did not improve nor deteriorate during the 54 h preceding the HOT, but improved drastically after the treatment, is a strong argument in favour of the beneficial effects of hyperbaric oxygen therapy.

## Conclusion

Post-operative cerebral arterial air emboli is a tricky, mimicking, underdiagnosed, underreported and rare entity with a high morbidity and mortality index. Therefore, the diagnosis should be made as early as possible with a high index of suspicion and solely on the clinical context (personal risk factor, surgery at risk, delayed awakening and/or neurological deficit). The neuro-imaging can exclude other diagnosis and orient but should not delay the initiation of hyperbaric oxygen therapy (HOT). In the absence of contraindications, assessing for patient stability and center accessibility are important factors that need to be part of the multidisciplinary discussion early on, in order to limit delay in management.

To this day, there is no randomised controlled trial or any other prospective study clearly setting a maximum delay for initiating HOT when having a suspicion of air emboli. However, for ethical reasons, and after a review of the literature, we advocate initiation of HOT, even with a delay over 48 h, whenever possible.

## Abbreviations

1 ATA: One atmosphere absolute (=760 mmHg baric pressure at sea level)
CPB: Cardiopulmonary Bypass
CT: Computed tomography
ECC: Extracorporeal circulation
EEG: Electroencephalogram
GCS: Glascow coma scale
GOS: Glasgow outcome scale
HOT: Hyperbaric oxygen therapy
ICU: Intensive care unit
MRI: Magnetic resonance imaging
VSD: Ventriculo-septal defect
